# SASP – Physiotherapy beyond 100 years of rehabilitation

**DOI:** 10.4102/sajp.v80i1.2135

**Published:** 2024-11-29

## Abstract

This conference report includes abstracts from the centenary congress held in Cape Town in September 2024, marking a significant milestone in the celebration of 100 years of the South African Society of Physiotherapy™ (SASP^®^). As part of the celebration, the SASP^®^ hosted the World Physiotherapy – Africa Region Congress, which brought together diverse international and African perspectives. The scientific programme had exciting sessions, focussing on physiotherapy and multidisciplinary aspects. Engaging Indaba and Debate sessions covered topics such as Strengthening Rehabilitation in Africa, Innovation in Rehabilitation, Reframing Care for People with Musculoskeletal Pain, Research in Children with Disabilities and Translating Research into Practice in Africa. These abstracts reflect the broad range of research and ideas presented, contributing to the future of physiotherapy and rehabilitation on the continent.

