# Supplementary material for: SASP – Physiotherapy beyond 100 years of rehabilitation
Source: S Afr J Physiother. 2024 Nov 29;80(1):2135. doi: 10.4102/sajp.v80i1.2135 (PMC11622128; doi:10.4102/sajp.v80i1.2135)
Supplement: Related texts

# Abstract: Research as a refinement tool for training and practice in medical rehabilitation: An Allied Health Professions Council (AHPC)-authorised conference

**Published:** 2024-11-29

**Authors:** Ajediran Idowu Bello, Nurudeen Tunde Amusat, Beatrice Sankah, Peter O. Ndaa, Gifty Nyante, Hosea Boakye

**Affiliations:** 1 Department of Physiotherapy, College of Health Sciences, University of Ghana, Accra, Ghana; 2 Department of Rehabilitation, Covenant Health, St Joseph General Hospital, Greenville, Alberta, Canada; 3 Department of Occupational Therapy, School of Biomedical and Allied Health Sciences, College of Health Sciences, University of Ghana, Accra, Ghana; 4 Department of Physical Therapy, Boston University, United States

## Abstract

**Background:**

Rehabilitation professions, including physiotherapy, occupational therapy, audiology, speech therapy and respiratory therapy, provide a viable healthcare continuum, yet the practitioners’ engagement in research remains paltry, particularly in sub-Saharan Africa.

**Method:**

A total of 193 participants, including physiotherapists, occupational therapists, respiratory therapists, audiologists, speech and language therapists, and optometrists, attended the 5-day online conference from both within and outside Ghana. The conference theme was ‘Building Skillsets for Research Engagement Among Ghanaian Rehabilitation Practitioners,’ and it was explored through five sub-themes. Experts from the rehabilitation professions facilitated each sub-theme. Following each presentation, a brainstorming session, which included questions and answers, was held. Reflective exercises were also assigned as take-home tasks, and feedback was provided to inform future endeavours.

**Results:**

The conference outcome highlighted the need for common ground in research engagement among clinicians and researchers. Proposed pragmatic measures include initiating practice-focussed questions, promoting participatory action research, engaging in research as a daily practice (READ-P) and repositioning practice plans to utilise research findings while collaborating to write for publication.

**Discussion:**

The knowledge update provided during the conference is expected to foster collaboration between rehabilitation clinicians and academics. It has established a platform for future partnerships, primarily between clinicians and researchers, thereby creating a promising pathway for harmonising the utilisation of research findings in practice.

**Conclusion:**

Discussion with participants and experts has further uncovered the real-time challenges hindering research engagement and the utilisation of its findings, while also suggesting pragmatic measures to address these issues in both the short and long term.

**Clinical implications:**

The conference’s outcome has implications for transdisciplinary, interdisciplinary and intradisciplinary research engagements, thereby ensuring better training and practice.

# Abstract: ‘It’s a matter of degrees’: Quantifying paediatric burns at Ghana’s leading burns centre

**Published:** 2024-11-29

**Authors:** Alberta Amissah Rockson, Sonti Pilusa, Natalie Benjamin-Damons

**Affiliations:** 1 Department of Physiotherapy, Faculty of Therapeutic Sciences, University of Witwatersrand, Johannesburg, South Africa; 2 Department of Physiotherapy, National Reconstructive Plastic Surgery and Burns Center, Korle Bu Teaching Hospital, Greater Accra, Ghana

## Abstract

**Background:**

Paediatric burns represent a significant public health concern in Ghana, yet this issue remains largely unaddressed, and there is a lack of data on its prevalence.

**Objectives:**

The objective of our study was to establish paediatric burns’ characteristics, prevalence, predictors and outcomes.

**Method:**

This retrospective cohort study analysed data from 608 patients who presented with burns from all over Ghana between 2020 and 2022. It comprised 335 paediatric patients aged 0–14 years. Descriptive analyses characterised the sample. Logistic regression and Chi-square tests examined predictors and associations with mortality, including age, sex, total body surface area (TBSA), length of stay (LOS) and reporting timelines.

**Results:**

The predominant patients were males (57.6%) aged 1–5 years (70.1%), with a mean TBSA of 22.12% and most causes of paediatric burns were from hot substances. The prevalence of paediatric burns was 50.6%, with patients reporting from several regions across Ghana. The mortality rate was 20.0%. In the multivariate logistic regression, higher TBSA (AOR 0.88) and longer LOS (AOR 1.07) were significant predictors of mortality (*p* < 0.001). Chi-square analysis found a significant association between higher TBSA category and mortality (*p* < 0.001).

**Conclusion:**

Our study found a high prevalence of paediatric burns, with hot substances being the predominant cause. Higher TBSA predicted worse survival. Older age and delayed presentation were marginally significant risks. Our study provided vital data on the national burden and epidemiology of paediatric burns presented at Ghana’s main burns treatment centre.

**Clinical implications:**

The findings have scientific value in informing prevention strategies, allocating resources and improving the quality of care.

# Abstract: Group exercise, self-efficacy education, and function in chronic lower back pain: Case report

**Published:** 2024-11-29

**Authors:** Ashlynn Wilkins, Emmarentia Wagner, Nina Sterrenberg, Jadee Kinnear

**Affiliations:** Department of Physiotherapy, Faculty of Medicine and Health Sciences, Stellenbosch University, Cape Town, South Africa

## Abstract

**Background:**

The high prevalence of lower back pain in Africa, along with its increasing chronic presentation, is placing a strain on low-resourced public healthcare services. The multifaceted nature of chronic lower back pain and the challenges surrounding effective treatment complicate management in these settings. This case report aimed to investigate the effects of a 6-week group exercise and education class on self-efficacy and physical function in an adult female with chronic lower back pain in a low-resource setting in South Africa.

**Method:**

This case study was conducted in a peri-urban community in the Western Cape. A 28-year-old female with chronic low back pain since a motor vehicle accident in 2018 was enrolled in a 6-week group exercise and education intervention. Patient-reported outcome measures focussed on pain intensity, function, and self-efficacy. The group education included pain neuroscience education and exercises, which encompassed aerobic dance, strength training and flexibility exercises.

**Results:**

Clinically meaningful improvements were seen in all patient-reported outcome measures, indicating improved pain self-efficacy and decreased functional limitations associated with chronic lower back pain.

**Conclusion:**

Exercise combined with pain neuroscience education is a low-resource investment that can lead to meaningful changes in self-efficacy, pain intensity and physical functioning. The detailed description of the intervention facilitates repeatability in larger samples or different settings, making it a valuable consideration for future research.

**Clinical implications:**

Exercise and education in a group format can be used in low-resource settings to enhance self-efficacy, reduce pain intensity and improve function.

# Abstract: Comparison of self-management versus physiotherapist-supervised protocols for patients with lumbar radiculopathy: A randomised controlled trial

**Published:** 2024-11-29

**Authors:** Bashir Bello, Maryam Abdulhadi, Leepile Sehularo, Ushotanefe Useh

**Affiliations:** 1 Department of Physiotherapy, Faculty of Allied Health Sciences, Bayero University Kano, Kano, Nigeria; 2 Department of Lifestyle Diseases Research Focus Area, Faculty of Health Science, North-West University, Mafikeng, South Africa

## Abstract

**Background:**

Lumbar radiculopathy (LR) significantly contributes to the global disease burden, particularly in cases of chronic low back pain. It is increasingly prevalent among older adults, young adults, and the working-class population, leading to substantial economic implications for society. Self-management therapy (SMT) has emerged as a potential management technique, empowering patients to take an active role in managing their condition. However, the comparative effectiveness of SMT and physiotherapy-guided management (PGMT) in treating LR has not been thoroughly investigated.

**Objectives:**

Our study aimed to address this gap by comparing the effectiveness of SMT and PGMT in managing LR.

**Method:**

Our study involved 100 participants diagnosed with LR who participated in a double parallel-arm randomised controlled trial. The participants were assessed using various measures, including pain intensity, functional disability, fear avoidance beliefs, quality of life and patient satisfaction.

**Results:**

The results indicated that the SMT group demonstrated greater improvement in pain intensity, functional disability, fear-avoidance belief and quality of life ([3 ± 0.2 vs 5 ± 0.9], [41.98 ± 8.5 vs 36 ± 10], [8.5 ± 1.5 vs 14 ± 2.8]) respectively, both at 8 weeks and at 6 months follow up (*p* < 0.001).

**Conclusion:**

In conclusion, our study provided evidence supporting the greater effectiveness of SMT over PGMT in managing individuals with LR.

**Clinical implications:**

These findings contribute to the growing body of evidence on the potential effectiveness of SMT in alleviating pain and enhancing functional disabilities in LR patients.

# Abstract: A scoping review of exercise programmes and adherence strategies used in the management of hand osteoarthritis

**Published:** 2024-11-29

**Authors:** Beatrice E.A. Sankah, Maria Stokes, Jo Adams

**Affiliations:** 1 School of Health Sciences, University of Southampton, Southampton, United Kingdom; 2 Department of Physiotherapy, College of Health Sciences, University of Ghana, Accra, Ghana

## Abstract

**Background:**

People with hand osteoarthritis (OA) often experience pain that limits their ability to perform daily functional tasks. While various exercises are available for managing hand OA, the extent of evidence-based exercises aimed at improving the quality of life for individuals with hand OA and supporting their informed management is not clearly defined.

**Objectives:**

To identify the strengthening and ROM exercises commonly used for hand OA management.

**Method:**

A scoping review was conducted according to the JBI (Joana Briggs Institute) scoping review methodology and reported according to the PRISMA extension for Scoping Reviews. Key findings were synthesised narratively.

**Results:**

Thirty-three records were included in our review. Regarding exercises for hand OA, the use of strengthening and range of movement (ROM) exercises was frequently reported as consistent with hand OA guideline recommendations. Common exercise adherence strategies used were telephone follow-up calls and exercise diaries.

**Conclusion:**

Six strengthening and ROM exercises commonly used for hand OA management are: (1) ‘making the O sign’, (2) making a fist, (3) finger and thumb stretch, (4) grip strengthening, (5) pinch strengthening and (6) thumb extension and abduction with elastic bands.

**Clinical implications:**

Based on the highlighted benefits and minimal related adverse effects, the inclusion of these six exercises in hand OA programmes is proposed, following further investigations on their effectiveness when used collectively in an exercise programme. A combination of telephone follow-up calls and exercise diaries is recommended to ensure adherence to these exercises.

# Abstract: Effect of workstation modifications: A low-cost strategy to prevent low back pain in the weaving sector in Burkina Faso

**Published:** 2024-11-29

**Authors:** Schepens Bénédicte, Kaboré A. Pegdwende

**Affiliations:** 1 Institute of Neuroscience, Faculty of Movement and Rehabilitation Sciences, UCLouvain, Louvain-la-Neuve, Belgium; 2 Centre National d’Appareillage Orthopédique du Burkina, Ougadougou, Burkina Faso

## Abstract

**Background:**

Work-related musculoskeletal disorders are a major health issue for low-income countries because of their high prevalence among workers and the lack of preventive health measures. In Burkina Faso, low back pain is the most prevalent disorder among weavers, with an annual prevalence of 71% to 77%.

**Objectives:**

The objective of our study was to assess the effect of workstation modifications on lumbar constraints.

**Method:**

A quantification of the compression forces applied to the lumbar disk (L5-S1) was performed on 25 weavers using 3D Static Strength Prediction Program (3DSSPP) before and after modifications to the workstation layout aimed at reducing low back flexion (during yarn preparation), the loads carried (during dyeing) and prolonged sitting (during weaving). Student’s paired t-tests were used for analysis. Additionally, correlations between compression forces, age, and body mass index (BMI) were examined using Pearson correlations.

**Results:**

Compression forces on L5-S1 were significantly decreased for each sub-task with a 27%–64% variation rate. Compression forces were not correlated with the age of the participants but with their BMI before and after layout modifications.

**Conclusion:**

Low-cost modifications based on ergonomics rules proved to reduce the constraints on the low back region. Special attention must be paid to the BMI of the weavers.

**Clinical implications:**

Our study will benefit researchers and health professionals dealing with assessing occupational risks, preventing work-related musculoskeletal disorders (WRMSDs) and implementing care guidelines. Based on these results, an awareness-raising brochure has been developed to be distributed to weavers, expanding the focus on low back pain prevention to include workstation layout and primary health promotion.

# Abstract: Patient-centred primary healthcare: Physiotherapists’ perspectives on providing effective care for high-impact chronic pain in the Western Cape, South Africa

**Published:** 2024-11-29

**Authors:** Brett J.N Mason, Romy Parker, Martha Geiger

**Affiliations:** 1 Department of Anaesthesia and Perioperative Medicine, Faculty of Health Sciences, University of Cape Town, Cape Town, South Africa; 2 Centre for Disability and Rehabilitation Studies, Global Health, Faculty of Medicine and Health Sciences, Stellenbosch University, Cape Town, South Africa; 3 Division of Disability and Rehabilitation Studies, Faculty of Medicine and Health Sciences, Stellenbosch University, Cape Town, South Africa

## Abstract

**Background:**

In South Africa, a significant portion of the population (83%) relies on public healthcare services, primarily accessed through primary healthcare (PHC) facilities. Individuals experiencing high-impact chronic pain (HICP) frequently visit PHC facilities because of participation restrictions and associated disabilities, which add a substantial burden to the healthcare system. In rural PHC settings, a single physiotherapist is often expected to manage HICP with limited support. Despite the presence and practice of physiotherapists in PHC, many individuals with HICP continue to endure the associated disabilities.

**Objectives:**

This qualitative descriptive study aimed to identify the barriers and facilitators reported by physiotherapists in Western Cape PHC facilities.

**Method:**

The qualitative descriptive study utilised semi-structured interviews with eight purposively selected physiotherapists from Western Cape PHC facilities, and inductive thematic analysis was implemented to interpret the data.

**Results:**

The findings revealed that participants perceived the complexity and disability associated with HICP as a burden that overwhelms them. They identified patient-centred approaches and collaborative strategies as facilitators to care. However, barriers to providing effective care included time constraints, patient-related factors, systemic issues, environmental conditions and physiotherapist-related challenges. Physiotherapists noted that their needs pertained to issues of time, knowledge and support.

**Conclusion:**

Our study concludes that HICP presents a significant challenge in South African PHC, highlighting the need for a patient-centred and collaborative approach to manage this condition. Overcoming the multifaceted barriers to effective care requires resources that enhance physiotherapists’ time, support and knowledge.

**Clinical implications:**

These resources may include a focus on innovative practices, networking with existing support structures, increasing the number of physiotherapists in PHC settings, employing rehabilitation-proficient managers and providing pain-specific training for South African PHC professionals.

# Abstract: The use of active video and computer game technology in physiotherapy practice: Perceptions of the physiotherapist – A qualitative study

**Published:** 2024-11-29

**Authors:** Bridget Numarce, Heather Weber, A. Lim, Kwawukume-Narh, Maayken van den Berg

**Affiliations:** 1 Department of Physiotherapy, University of Ghana, School of Biomedical and Allied Health Sciences, Accra, Ghana; 2 College of Nursing and Health Sciences, Flinders University, Adelaide, Australia; 3 Department of Physiotherapy, Lekma Hospital, Ghana Health Service, Teshie-Accra, Ghana; 4 Department of Medicine, Family Health Medical School, Family Health University College, Teshie-Accra, Ghana

## Abstract

**Background:**

Active video and computer (AVC) games technology is an emerging trend in rehabilitation that has been trialled with reported efficacy in stroke, spinal cord injury, cerebral palsy and brain injury rehabilitation. However, the physiotherapist’s role in its use and design, and its effects on therapy delivery have not been clearly demonstrated in most studies.

**Objectives:**

To explore the perceptions of physiotherapists’ use of AVC games technology and its effect on the mode of therapy delivery in rehabilitation.

**Method:**

Qualitative exploratory study with semi-structured interviews of eleven physiotherapists who used AVC games in rehabilitation. Perspectives on the role they play in AVC games were explored using thematic analysis. Interviews were recorded, transcribed and imported into NVivo for analysis.

**Results:**

The four major themes emerging from the perspectives of physiotherapists were: active video and computer game technology as an interactive and beneficial tool, pitfalls of active video and computer game technology, active video and computer game technology as a double-edged sword, and active video and computer game technology as a ‘third wheel’ between the patient and the physiotherapists.

**Conclusion:**

Overall, AVC game technology was perceived as a beneficial adjunct to therapy in facilitating the achievement of rehabilitation goals in a stimulatory, interactive and fun way.

**Clinical implications:**

Active video and computer games technology presents an enriching and beneficial platform for increased uptake of rehabilitative therapies. However, the quality of clinical benefits is not fully realised unless physiotherapists are actively involved in their use, design and innovatively tailor these benefits to achieving rehabilitation outcomes.

# Abstract: Policy analysis of a University mental health policy: Implications for rehabilitation students

**Published:** 2024-11-29

**Authors:** Naeema A.R Hussein El Kout, Raeesah Wadee, Bryce Till, Nare H. Shadung

**Affiliations:** Department of Physiotherapy, School of Therapeutic Sciences, Faculty of Health Sciences, University of the Witwatersrand, Johannesburg, South Africa

## Abstract

**Background:**

The prevalence of mental health conditions is increasing both globally and in South Africa. The implications of mental health for tertiary institutions are significant, as 15% of university students experience mental health impairments, which can lead to challenges in academic performance and, consequently, affect their ability to contribute to society. Therefore, university policies require critical analysis to identify gaps and ensure inclusivity.

**Objectives:**

Our study aimed to review the actors, content, context and processes of the student mental health policy at a tertiary institution in South Africa.

**Method:**

Our study ensured a participatory action research approach by including university-going students throughout our study. A document review was employed to review the Student Mental Health Policy at a tertiary education institution.

**Results:**

Data were analysed qualitatively on Maxqda through deductive thematic analysis, with pre-determined themes being the dimensions of the framework, namely, the content, process, actors and context from the Walt and Gilson policy triangle framework.

**Conclusion:**

While individual aspects of the policy were commendable, the policy lacked integration between actors, content, context and processes.

**Clinical implications:**

The lack of end-user engagement resulted in malalignment to global mental health agendas. Further research is required to develop strategies to address mental health challenges for university-going students.

# Abstract: Aetiology and motor function in post-neonatal cerebral palsy: Findings from the Northern Ireland cerebral palsy register over three decades

**Published:** 2024-11-29

**Authors:** Claire Kerr, Oliver Perra, Karen McConnell, Therese Kearney, Róisín Keenan

**Affiliations:** School of Nursing and Midwifery, Queen’s University Belfast, Belfast, United Kingdom

## Abstract

**Background:**

Postneonatal cerebral palsy (CP) describes a non-progressive brain injury occurring between 28 days and 2 years. It accounts for a third of CP cases in low- and middle-income countries. Still, less than 10% in high-income countries indicate a role for preventive measures that may reduce demand for future rehabilitation services.

**Objectives:**

Our study investigated postneonatal CP causes, frequency changes over time and differences in motor ability between postneonatal and perinatal CP cases.

**Method:**

Participants were *n* = 124 individuals with postneonatal CP, born 1981–2017, known to the Northern Ireland CP Register (6% of all cases; 95% CI: 5% to 7%). The Surveillance of Cerebral Palsy in Europe system was used to categorise postneonatal events; motor ability was categorised using the Gross Motor Function Classification System (GMFCS). Descriptive analysis of cross-sectional data for frequencies and logistic regression for dichotomised GMFCS (levels I–III vs. levels IV and V) were undertaken.

**Results:**

Infections were the most common primary cause (*n* = 44, 35% of postneonatal cases; 95% CI: 26% to 48%), presenting higher numbers in the 80s than the 90s or 00s (42%, 32%, and 29%, respectively). Postneonatal cases displayed increased odds for GMFCS categories IV or V (OR = 1.47; 95% CI: 1.01 to 2.15).

**Conclusion:**

Decreases in infection as a cause of postneonatal CP likely reflect healthcare improvements in recent decades. Children with postneonatal CP are at increased risk of poorer functional outcomes.

**Clinical implications:**

Further surveillance and preventive measures, such as rehabilitative interventions for children with postneonatal CP, are warranted.

# Abstract: Activity preferences of children with cerebral palsy and non-disabled peers in Saudi Arabia

**Published:** 2024-11-29

**Authors:** Haifa Alfouzan, Claire Kerr, Sonya Clarke, Malcolm Brown

**Affiliations:** School of Nursing and Midwifery, Queen’s University Belfast, Belfast, United Kingdom

## Abstract

**Background:**

Cerebral palsy (CP) is a lifelong condition often resulting in reduced physical activity. Children with CP face challenges in participating in activities of daily living and physical activities; however, their preferences for participation are not well described.

**Objectives:**

Our study describes activity preferences in children with CP and their non-disabled peers in Saudi Arabia.

**Method:**

Participants were 183 children (60 with CP, 123 non-disabled) aged 5–12 years from two geographically defined areas of Saudi Arabia. Children with CP were recruited via physiotherapists; non-disabled peers were recruited through four primary schools. A cross-sectional survey using the Preferences for Activity of Children (PAC) questionnaire was conducted. Activity preferences were categorised as recreational, physical, social, skill-based or self-improvement. Data were analysed descriptively, and the Chi-square test assessed differences between groups.

**Results:**

Children with CP showed a strong preference for recreational (mean 2.98/3; preferred by 60%) and social activities (mean 2.93/3; 40%). Conversely, non-disabled children exhibited a higher mean preference for physical activities (mean 2.93/3; 30%) followed by social (mean 2.89/3; 30%) and recreational activities (mean 2.89/3; 20%). Significant differences between the groups were observed only in preference for physical activities (*p* < 0.05).

**Conclusion:**

Understanding children’s individual activity preferences is crucial for designing effective physical activity interventions. Further exploration of clinical and environmental factors influencing activity preferences is needed.

**Clinical implications:**

Healthcare professionals should develop personalised intervention plans that consider each child’s unique activity preferences. This tailored approach may enhance motivation and adherence to physical activity programmes.

# Abstract: Perceived facilitators and barriers to physical activity among postpartum women in a selected tertiary hospital in Accra, Ghana

**Published:** 2024-11-29

**Authors:** Dorothy Ekua Adjabu, Jonathan Quartey, Diphale J. Mothabeng

**Affiliations:** 1 Department of Physiotherapy, Faculty of Healthcare Sciences, University of Pretoria, Pretoria, South Africa; 2 Department of Physiotherapy, Faculty of Health Sciences, University of Ghana, Accra, Ghana

## Abstract

**Background:**

Physical activity during the postpartum period is essential to promote optimal physical and mental maternal health and prevent and manage non-communicable diseases. Despite these known benefits, there are challenges in the adoption of this habit.

**Objectives:**

Our study aimed to identify perceived facilitators and barriers to participating in physical activity within the 1-year postpartum period.

**Method:**

A qualitative study using purposive sampling was conducted at the child welfare clinic of the 37 Military Hospital in Accra, Ghana. Twenty postpartum women were interviewed using an interview guide. Interviews were transcribed verbatim and thematically analysed.

**Results:**

Three themes emerged as influencing factors in physical activity participation. These included individual (a lack of time, fear of injury, financial constraints, baby-related factors, health benefits, perception about exercise and a lack of information), environmental (inadequate resources and weather conditions) and socio-cultural (social support, health worker motivation and cultural perceptions) factors.

**Conclusion:**

Promoting a lifestyle of physical activity in the postpartum period can be achieved by tackling the concerns and enablers of physical activity.

**Clinical implications:**

Identifying the facilitators and barriers to physical activity participation among postpartum women in low-resource settings is crucial in formulating context-specific or culturally sensitive strategies to promote a lifestyle of physical activity in this group.

# Abstract: The effects of a physical activity programme on improving quality of life of recovering Nyaope users in Tshwane

**Published:** 2024-11-29

**Authors:** Moitheki E. Molapisi, Douglas M. Maleka, Kebogile E. Mokwena

**Affiliations:** 1 Department of Physiotherapy, Faculty of Healthcare Sciences, Sefako Makgatho University, Pretoria, South Africa; 2 Department of Public Health, Faculty of Healthcare Sciences, Sefako Makgatho University, Pretoria, South Africa

## Abstract

**Background:**

Substance abuse remains one of the most serious and devastating public health problems in South Africa, presenting multiple challenges. Notably, Nyaope profoundly affects the psychosocial well-being, quality of life and physical function of its users. Quality of life (QoL) refers to the general well-being of individuals and societies, encompassing both negative and positive aspects of life. It is closely related to a fundamental human desire: to live well and feel good. Several factors can interfere with this quality, including issues arising from substance use, which can in turn affect overall life satisfaction.

**Objectives:**

To determine the effects of Nyaope on the QoL of recovery users.

**Method:**

A convenience sampling technique was used to select the participants for the QoL questionnaire based on the inclusion and exclusion criteria. The questionnaire was filled out at the beginning and end of the physical activity programme. Stata was used to analyse data.

**Results:**

The results indicated that the physical activity programme has improved the QoL of recovering Nyaope users significantly.

**Conclusion:**

Exercising positively impacts QoL among recovering Nyaope users.

**Clinical implications:**

To make the physical activity programme part of rehabilitation of substance abuse at formal rehabilitation centres and step-down facilities in South Africa as it improves their QoL.

# Abstract: Knowledge, attitudes and practices of South African physiotherapists regarding the management of gynaecological cancer survivors

**Published:** 2024-11-29

**Authors:** Jane Welman, Anke van der Merwe, Mariëtte Nel, Roline Y. Barnes

**Affiliations:** 1 Department of Physiotherapy, Faculty of Health Sciences, University of the Free State, Bloemfontein, South Africa; 2 School of Health and Rehabilitation Sciences, Faculty of Health Sciences, University of the Free State, Bloemfontein, South Africa; 3 Department of Biostatistics, Faculty of Health Sciences, University of the Free State, Bloemfontein, South Africa

## Abstract

**Background:**

Gynaecological cancer (GC) accounted for 13% of all new cancer cases in South Africa in 2022. Various physiotherapeutic interventions can be utilised to improve side effects caused by GC treatment.

**Objectives:**

Our study aimed to describe South African physiotherapists’ knowledge, attitudes and practices regarding the management of GCSs.

**Method:**

An observational, cross-sectional study was conducted via an online self-developed knowledge, attitudes, and practices (KAP) survey among physiotherapists registered with the Health Professions Council of South Africa (HPCSA) in 2021.

**Results:**

Of the 130 participants, 90.7% were female and 9.3% male. The median age was 37 (IQR:30–47), 23.3% had managed GCs and 39.1% received some training in the Pelvic and Women’s Health Physiotherapy field. Fifty-four (41.5%) participants had poor knowledge regarding the management of GCs. All participants agreed that the role of physiotherapists as part of the gynaecological multidisciplinary oncology team is not well understood in South Africa. There was a significant difference in the knowledge (*p* < 0.01) and practices of participants that have managed GCs versus those that have not managed this population. A significant difference was found between participants’ knowledge and whether they attended Pelvic and Women’s Health courses in their lifetime (*p* < 0.01).

**Conclusion:**

Rehabilitation is crucial in GC survivorship care to improve the quality of life of these patients. South African physiotherapists managing GCs should upskill themselves in the Pelvic and Women’s Health Physiotherapy field to ensure the best care for GCs.

**Clinical implications:**

The role of physiotherapists as part of gynaecological multidisciplinary oncology teams in South Africa has to be promoted among all healthcare professionals.

# Abstract: Biomechanical profile of yoga postures commonly used in low back pain management

**Published:** 2024-11-29

**Authors:** Ildephonse Nduwimana, Jean-Louis Thonnard

**Affiliations:** 1 Institute of Neuroscience, Université catholique de Louvain, Brussels, Belgium; 2 National Institute of Public Health, Bujumbura, Burundi

## Abstract

**Background:**

Chronic low back pain (CLBP) is sometimes resistant to conventional interventions; therefore, alternate therapies such as yoga are increasingly being used. Yoga has been demonstrated to have many effects on people with low back pain (LBP). Many yoga postures have been used in LBP management, but less information is available regarding their biomechanical profiles.

**Objectives:**

Our review study aimed to analyse the biomechanical profiles of yoga postures commonly used in CLBP management.

**Method:**

A literature search for commonly used yoga postures in LBP management and their biomechanical profiles was performed using Cochrane Library, PubMed, Scopus, PsycINFO and ScienceDirect.

**Results:**

Sixty-one yoga postures, which have been used at least in one randomised controlled trial (RCT), have been identified through 25 RCTs. Studies using electromyography highlighted specific muscle activation in various yoga postures. In addition, a co-activation of the trunk and hip muscles has been observed during some specific yoga postures. However, some loading or compressive forces generated during yoga practice could be a source of injuries. That is why adaptations and modifications of some yoga postures, especially in people with some comorbidities, are needed.

**Conclusion:**

From the available evidence, biomechanical profiles of yoga poses provide helpful information regarding the choice of comprehensive, safe and specific yoga programme for CLBP management. However, the biomechanical profiles of some commonly used yoga postures are still underexplored. Therefore, further biomechanical-based studies are needed.

**Clinical implications:**

These findings should help clinicians and yoga therapists plan tailored, safe and comprehensive yoga programme for CLBP.

# Abstract: Inclusion in primary physical education from the perspectives of educational staff and pupils with disabilities: A qualitative systematic review

**Published:** 2024-11-29

**Authors:** Chloe Leydon, Kay Pedlow, Jean Daly-Lynn, Sinead Sinead, Karen McConnell

**Affiliations:** 1 Department of Doctoral College, Faculty of Life and Health Sciences, Ulster University, Belfast, United Kingdom; 2 Department of Physiotherapy, Faculty of Life and Health Sciences, Ulster University, Belfast, United Kingdom; 3 Department of Psychology, Faculty of Life and Health Sciences, Ulster University, Belfast, United Kingdom; 4 Department of Sport and Exercise, Faculty of Sport Sciences, Ulster University, Belfast, United Kingdom; 5 School of Nursing and Midwifery, Queens University, Belfast, Northern Ireland

## Abstract

**Background:**

Physical education (PE) is vital to the health of all pupils. However, exclusion of pupils with disabilities is prevalent within primary school PE. No systematic reviews have explored inclusive PE from the perspectives of primary educational staff and pupils with disabilities.

**Objectives:**

Our review aimed to synthesise literature on the barriers and facilitators to inclusive PE from the perspectives of primary school educational staff and pupils with disabilities. Findings will inform physiotherapists’ recommendations for inclusive PE practices.

**Method:**

This qualitative systematic review followed PRISMA protocols. Information sources included Medline, CINAHL, SPORTDiscus, ERIC, Physical Education Index, ProQuest, OpenGrey and Google Scholar. Methodological quality was assessed using the JBI critical appraisal checklist for qualitative research. Of 2819 studies retrieved from systematic searches, 31 were eligible to be included. Participants included primary educational staff and pupils with disabilities.

**Results:**

Findings support collaboration between education and health professionals to facilitate inclusive PE. Barriers to inclusive PE comprised educators feeling ill-prepared to include pupils with disabilities and pupils feeling unsafe during PE. Facilitators included peer tutoring, specific adapted PE training and detailed lesson plans. Future research should establish the resources required by educators to enable the development of inclusive PE lessons.

**Conclusion:**

Inclusion of pupils with disabilities requires a collaborative approach; primary school educators welcome professional advice and strategies from physiotherapists to enhance PE for pupils with disabilities.

**Clinical implications:**

Physiotherapists should support primary educational staff in adapting PE activities and the environment for pupils with disabilities.

# Abstract: An observational study of maximum inspiratory pressure variations in mechanically ventilated adult patients: A physiotherapy perspective

**Published:** 2024-11-29

**Authors:** Kelebogile Mogopodi, Nombeko Mshunqane, Silmara Hanekom

**Affiliations:** Department of Physiotherapy, Faculty of Health Sciences, University of Pretoria, Pretoria, South Africa

## Abstract

**Background:**

Inspiratory muscle weakness contributes significantly to difficulty in weaning mechanically ventilated patients, thus resulting in re-intubation and prolonged stays in the intensive care unit (ICU). Assessment of inspiratory muscle strength is not usually included as part of physiotherapy in mechanically ventilated patients.

**Objectives:**

The main objective was to measure the changes in maximum inspiratory pressure in mechanically ventilated patients.

**Method:**

A quantitative prospective observational study was used. Participants who were intubated, mechanically ventilated and haemodynamically stable were included. A multistage sampling method was used where stratified random sampling was used for selecting three private hospitals and their ICUs. Maximum inspiratory pressure (MIP) was measured from the inbuilt ventilator function. Data were collected using a data collection sheet and analysed using a quantitative analysis approach. A *p*-value of < 0.05 was considered statistically significant.

**Results:**

Our study showed that MIP decreased from day 1 measurement (-22.4; standard deviation [s.d.] = 9.29) to day 10 (-12.5; s.d. = 2.12). The PaO2 also decreases as the MIP decreases, day 1 PaO2 (105.07; s.d. = 48.49) and day 10 (58.5; s.d. = 2.12), *p* = 0.005.

**Conclusion:**

Our study concluded that there is a significant decrease in inspiratory muscle strength in mechanically ventilated patients, thus contributing significantly to ICU-acquired weakness.

**Clinical implications:**

Our study suggests that the ventilator inbuilt maximum inspiratory pressure measurement is a safe and reliable tool for measuring inspiratory muscle strength.

# Abstract: Effect of educational programme on the perception of pain on function after lumbar spinal surgery

**Published:** 2024-11-29

**Authors:** Kgabo D. Masoma, Nombeko Mshunqane, Nontembiso Magida

**Affiliations:** Department of Physiotherapy, Faculty of Health Sciences, University of Pretoria, Pretoria, South Africa

## Abstract

**Background:**

Pain perception is a multidimensional concept related to several associated symptoms. The symptoms are embellished in patients who had spinal surgery and demonstrate poor quality of life. The literature demonstrates patient pain education to improve function and pain after spinal surgery.

**Objectives:**

Our study aimed to establish the effect of an educational programme on the perception of pain on function after lumbar spinal surgery.

**Method:**

Our study employed a prospective experimental single-blinded randomised control trial to recruit patients who had undergone lumbar spinal surgery and were admitted to a private hospital in Tshwane between January 2020 and December 2020. Participants were randomly assigned to either an experimental group or a control group using computer-generated random numbers. Data were collected using a validated Keel STarT-back screening tool (SBST) and a Pain Catastrophising Scale (PCS). Both groups received standard physiotherapy treatment, while the intervention group also received an educational video. The intervention was conducted over 12 weeks. Outcome measurements were taken the day before surgery, on the first day after surgery, and every two weeks for 12 weeks. Continuous data were analysed descriptively, and inferential statistics were used for categorical variables.

**Results:**

The control group results for PCS and SBST were statistically significant compared to the intervention group (*p* = 0.001). Thus, the null hypothesis was accepted, confirming that the pain education programme in the form of videos did not change the perception of pain on function in our study population after the 12 weeks of intervention. The findings of our study cannot be generalised because the sample size was small.

**Conclusion:**

Using an educational video did not have statistically significant effects on outcomes of patients who had undergone lumbar spinal surgery. However, the results must be interpreted with caution as the sample size was small.

**Clinical implications:**

While the use of technology in patient care is encouraged, care should be exercised when managing pain post lumbar spinal surgery.

# Abstract: Outcomes of adults recovering from major thoracic trauma following myofascial release and active exercise therapy interventions: A quasi-experimental study

**Published:** 2024-11-29

**Authors:** Kyra L. Blewitt, Heleen Van Aswegen

**Affiliations:** Department of Physiotherapy, Health Sciences, University of the Witwatersrand, Johannesburg, South Africa

## Abstract

**Background:**

Thoracic trauma results in decreased pulmonary function and pain, which affects survivors’ quality of life (QoL). The effects of myofascial release and active exercise therapy on patients’ clinical outcomes are unknown.

**Objectives:**

To determine the outcomes of adults recovering from major thoracic trauma who received myofascial release and active exercise therapy interventions over 6 months after discharge.

**Method:**

Prospective, longitudinal, quasi-experimental repeated measures design. Consecutive sampling was used to recruit individuals with major thoracic trauma from a private trauma hospital. Outcomes assessed prior to hospital discharge and at 4 and 12 weeks included QoL, pain, physical activity, pulmonary function, muscle strength and thoracic range of motion (ROM). Physiotherapy intervention included heat therapy, intercostal muscles, diaphragm myofascial release breathing exercises and active thoracic cage ROM exercises. Descriptive and inferential statistics were used to summarise and compare data.

**Results:**

Most participants were male (*n* = 27/35; 77%). At 3-months thoracic ROM (left: *p* = 0.001; right: *p* = 0.004) and pulmonary function (maximum inspiratory pressure [MIP]: *p* = 0.004; peak expiratory flow rate [PEFR]: *p* < 0.001) and at 6-months pain (brief pain inventory [BPI]: *p* < 0.05; EuroQol 5 Dimension [EQ-5D]: *p* = 0.023), handgrip strength (*p* < 0.001) and physical activity changed significantly, as did QoL, compared to baseline.

**Conclusion:**

Myofascial release and active exercise therapy contribute to the recovery of those with major thoracic trauma.

**Clinical implications:**

Findings are encouraging and contribute to the limited evidence in this field.

# Abstract: Caregiver experiences of a facilitation programme for children with malnutrition

**Published:** 2024-11-29

**Authors:** Maria C. Ras, Anri Human, Carina A. Carina

**Affiliations:** 1 Department of Health, Faculty of Public Service, Brits District Hospital, Brits, South Africa; 2 Department of Physiotherapy, School of Health Care Sciences, Sefako Makgatho Health Sciences University, Ga-Rankuwa, Pretoria, South Africa; 3 Department of Physiotherapy, Faculty of Health Sciences, University of Pretoria, Pretoria, South Africa

## Abstract

**Background:**

Early identification of developmental delay in children diagnosed with malnutrition requires collaborative efforts from caregivers and multidisciplinary healthcare members. A lack of caregiver experience and skills regarding developmental milestones and facilitation has been reported.

**Objectives:**

To determine caregivers’ experiences and skills in implementing a standardised physical activity programme for children diagnosed with malnutrition in the Bojanala district.

**Method:**

This case series consisted of three caregivers and their four children (3–15 months; one caregiver had twins) admitted for malnutrition at a district hospital in South Africa. The four children presented with a median (IQR) age of 11.7 (8.9–13.3) months and Alberta Infant Motor Scale (AIMS) percentile ranking z-scores of 5.0 (5.0–15.0), weight-for-age of -3.0 (-3.0 to -2.5) and weight-for-height of -1.0 (-2.0 to -1.0). Non-probability, purposive sampling was used. Data were collected at baseline and once a month during follow-up for 3 months. All caregivers provided written informed consent.

**Results:**

The caregivers reported limited child development experience and demonstrated poor handling skills with their children. Caregivers were trained to implement an individualised facilitation programme and presented with improved handling skills and confidence post-intervention. Caregivers reported improved experience and skills and would recommend this facilitation programme to others. Despite continued gross motor developmental delay, the children improved their AIMS scores over 3 months.

**Conclusion:**

A home-based, caregiver-implemented facilitation programme, as part of early intervention initiatives for children with malnutrition, could be of clinical benefit within the South African context. Further exploration and larger sample sizes are required.

**Clinical Implications:**

A home-based caregiver facilitation programme has the potential to enhance children with malnutrition’s gross motor development and caregiver handling skills and confidence.

# Abstract: Towards developing a competency framework to guide responsive physiotherapy practice

**Published:** 2024-11-29

**Authors:** Marianne Unger, Marieke Mocke, Susan D. Hanekom

**Affiliations:** 1 Department of Physiotherapy, Faculty of Medicine and Health Sciences, Stellenbosch University, Cape Town, South Africa

## Abstract

**Background:**

Task shifting and skill mixing and the emergence of new healthcare professional roles are strategies promoted by the World Health Organization (WHO) to assist in addressing society’s (neglected) needs and thus demand competencies that extend beyond discipline expertise.

**Objectives:**

Our study aimed (1) to identify physiotherapy (PT) competencies as described globally and (2) to use the WHO Rehabilitation Competency Framework (RCF) to develop a conceptual framework to guide further development of a framework to guide practice and inform the training of future physiotherapists in any setting.

**Method:**

A document review was conducted. Competency statements from all documents sourced from 125 World Physiotherapy member countries were extracted onto an Excel spreadsheet and uploaded to Atlas.ti (v22) and thematically analysed using inductive analysis. Each rephrased competency was then assigned to the appropriate WHO RCF domain using the definitions from this framework to develop a conceptual framework for further use.

**Results:**

Twenty documents were included in our review. Within the Practice domain (882 competency statements), which related to client and therapeutic procedure, six themes and 25 sub-themes emerged; the Professional domain included 488 statements from which three themes and 12 sub-themes emerged; followed by 315 in the Management and Leadership domain; 216 statements in the Learning and Development domain; and only 100 statements in the Research domain.

**Conclusion:**

Our study produced a conceptual framework to develop a locally responsive physiotherapy curriculum; but moving forward, competencies for advancing the social justice agenda such as transdisciplinary collaboration, broader stakeholder engagement, entrepreneurship, innovation, creativity, systems and design-thinking, among others, need to be strengthened and/or considered.

**Clinical implications:**

The use of responsive curricula have the potential of producing physiotherapists that are not only clinically competent but are socially accountable.

# Abstract: Patients’ perspectives, pain and health-related quality of life in same-day discharge hip and knee arthroplasty

**Published:** 2024-11-29

**Authors:** Martin Harry van Heerden, Sandy Lord, Monique M. Keller

**Affiliations:** Department of Physiotherapy, School of Health Sciences, University of the Witwatersrand, Johannesburg, South Africa

## Abstract

**Background:**

International protocols for same-day discharge (SDD) following total hip (THA) and total knee arthroplasty (TKA) have recently been introduced in South Africa. Comparisons between recovery trajectories of THA and TKA patients in traditional length-of-stay models and SDD are limited. The impact of SDD related to pain, health-related quality of life and the patient’s perspective has yet to be studied in the South African context.

**Objectives:**

Our study explored, evaluated and compared these aspects following SDD THA and TKA.

**Method:**

A mixed-methods design was implemented with a collection of patient-reported outcome measures (PROMs) over 6 weeks (*n* = 109: 55 THA and 54 TKA) and concluded with semi-structured interviews (*n* = 24: 14 THA and 10 TKA). Participants scheduled for SDD THA and TKA who met our study criteria were selected through convenience sampling. Descriptive and inferential statistics were used and a thematic analysis of interview data was conducted.

**Results:**

There were significant improvements in PROMs by 6 weeks postoperatively in both groups. Significant between-group differences revealed persistent neuropathic pain (*p* = 0.536) and worse health-related quality of life scores (*p* = 0.102) in the TKA group. The themes identified highlight positive perspectives of SDD, emphasising unique challenges in the SDD TKA group. Findings indicate that patients undergoing SDD THA and TKA in South Africa produce results comparable to those in international studies.

**Conclusion:**

This research advocates for a holistic approach in SDD THA and TKA, emphasising patient-centric care, effective communication and preparation.

**Clinical implications:**

Our study contributes insights into this evolving clinical pathway and encourages further research.

# Abstract: Utilisation and delivery of rehabilitation services among persons with Parkinson’s disease: A scoping review

**Published:** 2024-11-29

**Authors:** Mary W. Agoriwo, Conran Joseph, Erika Franzén, Marianne Unger

**Affiliations:** 1 Department of Health and Rehabilitation Sciences, Faculty of Medicine and Health Sciences, Stellenbosch University, Cape Town, South Africa; 2 Department of Physiotherapy and Rehabilitation Sciences, University of Health and Allied Sciences, Ho, Ghana; 3 Department of Physiotherapy, Karolinska Institutet, Stockholm, Sweden

## Abstract

**Background:**

Although numerous studies have reported the benefits of rehabilitation for persons with Parkinson’s disease (PwPD), these services are still limited and underutilised even when patients exhibit problems that require rehabilitation.

**Objectives:**

Our review sought to describe the indications and patterns for referral for rehabilitation services and factors that facilitate or hinder the utilisation and delivery of rehabilitation services among PwPD.

**Method:**

A scoping review and a comprehensive literature search were performed across six databases. Studies published in English from January 2002 until December 2022 were applied as limiters. Tracking of reference and grey data sources was also conducted. Two reviewers conducted our study selection and data charting. A descriptive analysis was performed.

**Results:**

Twelve studies were included in our review; however, none were from Africa. Impairments and activity limitations were the leading indicators for rehabilitation among PwPD. Physiotherapy, occupational therapy and speech and language therapy or pathology were the most utilised rehabilitation services. Age, gender, income, race, disease stage, specific functioning problems, quality of life and care by a neurologist were the key predictors for referral to rehabilitation. The rate of rehabilitation service utilisation among PwPD ranged from 0.9% to 62.5%. The common barriers to rehabilitation service utilisation were a lack of referrals and limited rehabilitation units/professionals.

**Conclusion:**

Utilisation of rehabilitation services is generally low among PwPD, with scarcity of research from Africa.

**Clinical implications:**

The findings inform the need for adequate measures to improve access and use of rehabilitation services among PwPD and more research from the African continent.

# Abstract: The prevalence and profile of spinal cord injury in public rehabilitation units in Gauteng, South Africa

**Published:** 2024-11-29

**Authors:** Michael A. Alves, Mokgadi K. Mashola, Sonti Pilusa

**Affiliations:** Department of Physiotherapy, Faculty of Health Sciences, University of Witwatersrand, Johannesburg, South Africa

## Abstract

**Background:**

Rehabilitation plays an essential role post spinal cord injury (SCI). Admission to a specialised SCI unit for acute and rehabilitative care has many benefits. The Rehabilitation 2030 Call for Action by the World Health Organization has highlighted the need for ongoing surveillance and collection of rehabilitation data for persons with spinal cord injury (PWSCI). In South Africa, SCI surveillance data is scanty.

**Objectives:**

Our study thus sought to profile PWSCI receiving rehabilitation in Gauteng’s public rehabilitation units.

**Method:**

A retrospective medical record review was conducted. A task-specific data collection form was created and used. Data were analysed using descriptive and inferential statistics.

**Results:**

During the 2-year study period, 386 PWSCI records were analysed. The mean age was 36.90 years, with significant findings in the traumatic spinal cord injury (TSCI) (younger) and non-traumatic spinal cord injury (NTSCI) (older) cohorts. Out of the total, 69.95% of all participants were male, with females being significantly more likely to sustain an NTSCI. Assault and transport-related injuries were common causes of TSCIs, while infection-related causes were most common in the NTSCI cohort. Most injuries (58.86%) were complete and in the lower thoracic spine (39.92%). The average time to rehabilitation admission was 64.48 days, with a mean length of stay of 85.59 days. The mortality rate was 6.48%.

**Conclusion:**

Gauteng’s SCI profile differs from global trends, with the highest proportion of assault-related TSCI globally. Further, women were more likely to sustain an NTSCI – the only country to report this globally.

**Clinical implications:**

Ongoing, accurate and standardised SCI surveillance is crucial in guiding data-driven decision-making and allowing for tailored rehabilitation through accurate provision planning. It will also allow for targeted preventative programmes.

# Abstract: Descriptive cross-sectional survey to management practices for patients with hand osteoarthritis in South Africa: A pilot study

**Published:** 2024-11-29

**Authors:** Monique M. Keller, Minni Christy Thomas, Tshifhiwa Mukheli, Josslyn Sarah de Kock, Rendani Muvhumbe, Monya Motsele, Sumayya Bhyat, Nomusa Mngoma

**Affiliations:** 1 Department of Physiotherapy, Faculty of Health Sciences, University of Witwatersrand, Johannesburg, South Africa; 2 Department of Physiotherapy, Sizwe Tropical Diseases Hospital, Modderfontein, South Africa; 3 Department of Oral Health & Therapeutic Services, Gauteng Department of Health, Johannesburg, South Africa; 4 Department of Physiotherapy, Charlotte Maxeke Johannesburg Academic Hospital, Johannesburg, South Africa; 5 Department of Physiotherapy, Yusuf Dadoo Hospital, Krugersdorp, South Africa; 6 Department of Physiotherapy Far East Rand Hospital, Springs, South Africa; 7 Department of Physiotherapy, Private Practice, Johannesburg, South Africa; 8 Department of Physiotherapy, Queens University, Ontario, Canada

## Abstract

**Background:**

Hand osteoarthritis (OA) is a debilitating and highly prevalent condition. The problem is the lack of knowledge on the current physiotherapy (PT) and occupational therapy (OT) management practices in South Africa.

**Objectives:**

The objective was to determine the management practices of PTs and OTs for patients presenting with hand OA.

**Method:**

A cross-sectional descriptive design with a REDCap survey was completed by purposively sampled participants who provided informed consent to participate in the ethically approved study.

**Results:**

Thirty-one participants, including 20 OTs and 11 PTs, comprised the study group. Of these, 29 were female and two were male, with a mean age of 36.42 years (standard deviation [s.d.] = 8.69) and an average of 4.65 years of experience (s.d. = 1.98). The majority worked in Gauteng, with 19 participants (61.3%) employed in the government sector, while 12 (38.7%) were in public hospitals and 11 (35.5%) in private outpatient practices. Carpometacarpal thumb OA was the predominant diagnosis, reported by 16 participants (51.6%). The most frequently used assessment tools for diagnosis included pain evaluation (21 participants, 67.7%; 15 OTs), subjective assessment and hand function (20 participants, 64.5%; 15 OTs), area of symptoms (19 participants, 61.3%; 14 OTs) and assessment of deformity and goniometry (18 participants, 58.1%; 13 OTs). The most common treatments applied were exercise (22 participants, 71%; 15 OTs), pain education (20 participants, 64.5%; 14 OTs) and activity modification (16 participants, 51.6%; 15 OTs). Adjustment of grips used during daily activities and massage were also frequently utilised (16 participants, 51.6%; 12 PTs). Other treatments included splinting (15 participants, 48.4%; 15 OTs), kinesiotaping (13 OTs) and soft tissue treatment (10 OTs).

**Conclusion:**

Standardised assessments are not frequently used. There is a lack of exercise and pain education used by physiotherapists.

**Clinical implications:**

Determining the current management trends allows for future education and research towards best-evidence management.

# Abstract: Patient perceived barriers and facilitators to weight-bearing with a lower limb circular external fixator

**Published:** 2024-11-29

**Authors:** Megan D. Crouse, Monique M. Keller, Sandy Lord

**Affiliations:** Department of Physiotherapy, Faculty of Health Sciences, University of Witwatersrand, Parktown, South Africa

## Abstract

**Background:**

Circular external fixators are commonly used in orthopaedics to immobilise broken bones and correct deformities. Early weight-bearing enhances bone growth and improves patient outcomes. However, many patients do not fully weight-bear after surgery, indicating the presence of barriers that need to be addressed.

**Objectives:**

The objective was to find the barriers and facilitators to full weight-bearing with a circular external fixator.

**Method:**

Patients with lower-limb circular external fixators in Pretoria were invited to participate in a qualitative exploratory study using semi-structured interviews. The interviews were audio-recorded, transcribed verbatim and analysed using thematic content analysis. Data saturation was reached after nine interviews.

**Results:**

Four themes were generated. These included pain and pain management, participants’ state of mind, physical factors and medical/non-medical support systems. Pain, swelling and the history of the injury influenced the participants’ ability to weight-bear and their state of mind. Physical factors relating to the human body and external fixator equipment also played a role, as did the availability of medical and non-medical support systems. Complications and fear of re-fracturing or falling were additional factors affecting mobilisation and weight-bearing.

**Conclusion:**

Our study findings are consistent with the limited existing literature on external fixators and provide valuable insights into the barriers and facilitators of full weight-bearing. They emphasise the importance of addressing physical and psychological factors.

**Clinical implications:**

The barriers and facilitators identified in our study should provide a better understanding among the clinicians managing these patients. They can be used as a guideline for the implementation of solutions.

# Abstract: Document review of the paper-based implementation of the framework and strategy for disability and rehabilitation in South Africa

**Published:** 2024-11-29

**Authors:** Naeema AR Hussein El Kout, Natalie Benjamin-Damons, Sonti Pilusa

**Affiliations:** Department of Physiotherapy, School of Therapeutic Sciences, Faculty of Health Sciences, The University of the Witwatersrand, Johannesburg, South Africa

## Abstract

**Background:**

The prevalence of disability is on the rise globally and in South Africa, with a high number of unmet needs and poor actualisation of the health rights of persons with disability. A tool to realise these rights is health policy, such as the framework and strategy for disability and rehabilitation (2022) (FSDR). There is limited data on the implementation outcomes for the FSDR.

**Objectives:**

Our study reviewed the implementation of the FSDR according to the paper-based provincial reports.

**Method:**

A document review of eight provincial paper-based implementation reports on the FSDR was conducted. Quantitative data were analysed using descriptive statistics. Qualitative data were analysed via deductive thematic analysis using Maxqda. The RE-AIM framework was used to categorise the findings according to adoption, maintenance, efficacy implementation and reach.

**Results:**

About 87% of provinces reported physical accessibility to the FSDR, and 62% of provinces received training on implementing the FSDR. Only two out of eight provinces have conducted monitoring and evaluation since implementing the FSDR in 2015. Qualitative findings revealed poor reach and adoption of the FSDR because of a lack of implementation training for end users. The lack of indicators resulted in poor maintenance of the FSDR and the lack of human resources and equipment, which resulted in reduced efficacy of the FSDR.

**Conclusion:**

The FSDR has not achieved its full level of implementation because of numerous barriers, such as the lack of resources, human capacity and training for implementation.

**Clinical implications:**

This may inform future implementation of disability policy to improve rehabilitation access.

# Abstract: Physical activity and exercise behaviours in adults living with a stoma, and the factors influencing their participation

**Published:** 2024-11-29

**Authors:** Naila Edries, Meg Davidson, Jamie Hollick, Sange Mjindi, Nonjabulo Sibiya, Tyra Woods

**Affiliations:** Department of Health and Rehabilitation Sciences, Faculty of Health Sciences, University of Cape Town, Cape Town, South Africa

## Abstract

**Background:**

A stoma is a surgical opening created on the abdomen to divert faeces from the bowel to relieve an obstruction caused by health conditions such as colorectal cancer or bowel disease. Despite the known benefits of physical activity, few studies have reported substantially reduced physical activity levels in persons living with a stoma.

**Objectives:**

To provide insight into the South African context, our study sought to explore the physical activity behaviours of adults living with a stoma and to determine the factors influencing their participation.

**Method:**

A quantitative, descriptive, cross-sectional study was conducted. Adults living with a stoma were invited to complete an online survey. The South African Society of Ostomates assisted with sharing the survey link. The International Physical Activity Questionnaire was used to determine the physical activity behaviours of participants. A self-developed questionnaire explored the factors influencing their participation.

**Results:**

Data analysis included 68 participants (*n* = 68), predominantly 1–5 years post colostomy. Most participants (58.8%) did not meet the American College of Sports Medicine’s recommended physical activity guidelines. Approximately 45% of the participants were not engaging in any physical activity. Stoma-related fears (leakage and hernia) and insufficient knowledge of exercise were identified barriers to participation.

**Conclusion:**

Our study found that persons living with a stoma in South Africa are not engaging in adequate levels of physical activity, influenced by a lack of knowledge.

**Clinical implications:**

Our findings highlight a need to promote physical activity in persons post colostomy, ileostomy and urostomy and empower them to exercise safely. A future study with a larger sample size should corroborate our results.

# Abstract: Change in severity of pelvic floor dysfunction in females three months after sustaining pelvic fractures: A reason for screening

**Published:** 2024-11-29

**Authors:** Nazreen Chopdat, Corlia Brandt

**Affiliations:** Department of Physiotherapy, Faculty of Health Sciences, University of the Witwatersrand, Johannesburg, South Africa

## Abstract

**Background:**

Early identification and physiotherapy intervention of pelvic floor dysfunction (PFD) are important to improve quality of life (QoL). Research is sparse on the occurrence and contributing factors of PFD in females post pelvic fracture in South Africa. Pelvic fractures are a risk factor for pelvic floor dysfunction.

**Objectives:**

The study aimed to determine PFD symptoms and associated factors among females 3 months post pelvic fracture compared to preinjury symptoms.

**Method:**

A 3-month quantitative longitudinal study was conducted of all females from Chris Hani Baragwanath Academic Hospital/Charlotte Maxeke Johannesburg Academic Hospital, aged 18 years or older, not pregnant and more than 4 months postpartum, recruited within 3 months of a pelvic fracture. Patient medical records and symptoms were collected through a questionnaire and the Australian Pelvic Floor Questionnaire (APFQ). Correlation tests and linear regression analysis were used.

**Results:**

The injury limited sexual activity in 45.45% of participants. Significant change was noted in Total (*p* = 0.0216), Bladder (*p* = 0.0062) and Sexual (0.0087) APFQ scores from preinjury to 3 months post-injury; and between acute and 3 months post APFQ Total (*p* = 0.0361), Bladder (*p* = 0.0002) and Bowel (*p* < 0.0001) scores. Prolonged urinary catheter usage increases the risk for high bladder PFD scores (*n* = 37, r = 0.1585). Factors associated with less PFD at 3 months include a number of vaginal deliveries preinjury and bedrest with non-weight bearing choice of treatment (*p* = 0.046, coef = -4.00, 95% CI: -7.92 to -0.08).

**Conclusion:**

Pelvic floor dysfunction is present prior to and at 3 months post pelvic fracture, with an increase in frequency and severity at 3 months. Symptoms are most prevalent in the acute phase. The results of our study support the relevance of screening for pelvic floor dysfunction within the acute phase and at 3 months for females following pelvic fractures.

**Clinical implications:**

Screening for PFD should become standard practice for females post pelvic fracture both in the hospital and during outpatient follow-ups. The duration of catheterisation should be justifiable. Screening for bladder dysfunction is pertinent in patients requiring prolonged catheterisation and patients requiring less conservative management. Guidance on safety to return to sexual activity is important to include as part of routine follow-up orthopaedic sessions.

# Abstract: Digitalisation in physiotherapy – An African perspective

**Published:** 2024-11-29

**Authors:** Olebogeng T. Masilo, Njabulo F. Mnisi, Ntsako A. Maluleka, Shanquelle T. Mokase, Cilia N. Phalane, Rhoda Mwepu, Maria E. Cochrane

**Affiliations:** 1 Department of Physiotherapy, School of Health Care Sciences, Sefako Makgatho Health Sciences University, Ga-Rankuwa, South Africa; 2 Department of Health Professions Education, Faculty of Health Sciences, University of Pretoria, Pretoria, South Africa

## Abstract

**Background:**

The Fourth Industrial Revolution has changed how healthcare is delivered to patients. A comprehensive overview of how physiotherapists in Africa are implementing digitalisation when managing patients is not available.

**Objectives:**

To establish the level of digitalisation uptake among physiotherapists in Africa.

**Method:**

A narrative review study design was undertaken. Literature was included if published within the last 10 years, in English, and if the studies were conducted in an African country. All published methodologies were considered for inclusion. Joana Briggs Institute (JBI) critical appraisal tools were used to ensure the quality of the included literature.

**Results:**

A total of 625 articles were identified through the various databases searched. Screening for relevance and eligibility yielded 102 potential articles for inclusion. All 102 articles were read and appraised; ultimately, 15 were included in our review.

**Conclusion:**

Digitalisation uptake among physiotherapists in Africa is sporadic and poorly researched. Some physiotherapists employ robotic process automation for patient booking and management, but the use is inconsistent. Telehealth is not as frequently used as anticipated and has the potential to change how services are provided to patients, especially in rural areas.

**Clinical implications:**

There is no evidence that African physiotherapists use artificial intelligence, big data, additive manufacturing and biotechnology.

# Abstract: Acceptability of telerehabilitation among stroke survivors in Nigeria

**Published:** 2024-11-29

**Authors:** Olubukola A. Olaleye, Ayomide D. Adepoju

**Affiliations:** 1 Department of Physiotherapy, Faculty of Clinical Sciences, University of Ibadan, Ibadan, Nigeria; 2 Department of Physiotherapy, Lead City University, Ibadan, Nigeria

## Abstract

**Background:**

Telerehabilitation has emerged as a pragmatic solution to challenges posed by the scarcity of providers and resources, as well as enhancing access to rehabilitation services. Nonetheless, to ensure the acceptability of telerehabilitation, it is crucial to investigate user perceptions and lived experiences.

**Objectives:**

We explored the acceptability of telerehabilitation among stroke survivors in Ibadan, Nigeria.

**Method:**

Using a mixed-method approach, 44 stroke survivors consecutively recruited from physiotherapy clinics in Ibadan were surveyed, and a focus group discussion (FGD) was held with six participants. Quantitative data were analysed using inferential statistics at α = 0.05, while qualitative data were thematically content analysed.

**Results:**

Participants, predominantly males (61.4%), were aged 60.9 ± 13.1 years. Results showed a significant relationship between age and acceptability (*p* < 0.05). Acceptability of telerehabilitation as an effective tool for treatment differed across marital status, level of education and socioeconomic status (*p* < 0.05). Participants in the FGD viewed telerehabilitation positively but were hesitant to embrace it as a primary treatment option. Barriers to acceptability included unstable Internet connectivity, high costs of data bundles and the absence of personal touch from physiotherapists. Facilitators included government support for equipment and data provision and using telerehabilitation for make-up sessions for missed appointments.

**Conclusion:**

While participants in our study viewed telerehabilitation positively, they preferred it as a complementary tool rather than a replacement for traditional face-to-face physiotherapy.

**Clinical implications:**

Even though telerehabilitation benefits underserved communities, patients still prefer the human connection of traditional face-to-face physiotherapy. Thus, telerehabilitation should be supplementary rather than a one-size-fits-all approach in physiotherapy.

# Abstract: Participation of fathers/partners and siblings in home rehabilitation programmes for children with neuro-developmental delay: A scoping review

**Published:** 2024-11-29

**Authors:** Precious Madzimbe, Soraya Maart, Lieselotte Corten, Jermaine Dambi

**Affiliations:** 1 Department of Health Rehabilitation Sciences, Faculty of Health Sciences, University of Cape Town, Cape Town, South Africa; 2 Department of Physiotherapy and Occupational Therapy, United Bulawayo Hospitals, Bulawayo, Zimbabwe; 3 School of Education, Sport and Health Sciences, University of Brighton, Brighton, United Kingdom; 4 Department of Rehabilitation Sciences, Faculty of Medicine and Health Sciences, University of Zimbabwe, Harare, Zimbabwe

## Abstract

**Background:**

The role of, and impact on, mothers caring for children with neuro-developmental delay (NDD) continues to receive significant clinical and research interest. However, the role of fathers/partners and siblings in families of children with NDD remains significantly understudied, particularly in low and middle-income countries (LMICs). There has been an increased call for holistic rehabilitation of children with NDD at the family level.

**Objectives:**

Our study aimed to explore the involvement of fathers/partners and siblings in the home rehabilitation programmes of children with NDD.

**Method:**

A scoping review was conducted in accordance with the PRISMA-ScR guidelines. Peer-reviewed articles were retrieved from PUBMED, ScienceDirect, PsycINFO, Africa Wide, SCOPUS and PEDro. Google Scholar was used to access grey literature. Additionally, the reference lists of relevant studies were manually searched.

**Discussion:**

Twenty-nine peer-reviewed articles were identified. Father/partner and sibling participation in home rehabilitation programmes and caregiving is low in LMICs compared to high-income countries because of economic factors, and deeply rooted religious and cultural beliefs. Reduced father/partner and sibling participation in home rehabilitation programmes is stressful to mothers and reduces developmental outcomes in children with NDD.

**Conclusion:**

This scoping review demonstrates the need for rehabilitation professionals to encourage father/partner and sibling participation in the caregiving of children with NDD in home rehabilitation programmes.

**Clinical implications:**

A different approach by rehabilitation professionals that engages fathers/partners and siblings in rehabilitation home programmes is recommended as it will potentially alleviate stress and physical burden on mothers as well as improve developmental outcomes in children with NDD.

# Abstract: Prevalence and associated factors of musculoskeletal disorders among health professional students at Malaysia University of Science and Technology

**Published:** 2024-11-29

**Authors:** Priscillah Ondoga, Janet R. Namara, Tresor Ntwari, Joshua Olinga, Japheth Ainomugisha, Jean Damascene Niyonsenga

**Affiliations:** 1 Department of Physiotherapy, Faculty of Medicine, Mbarara University of Science and Technology, Mbarara, Uganda; 2 Department of Physiotherapy, Ropheka Physiotherapy, Kampala, Uganda; 3 Department of Physiotherapy, Faculty of Medicine, Mbarara Regional Hospital, Mbarara, Uganda

## Abstract

**Background:**

About 1.7 million health professional students live with musculoskeletal disorders (MSDs) globally.

**Objectives:**

The study aimed to establish the prevalence and associated risk factors of MSDs among health professional students at Malaysia University of Science and Technology (MUST).

**Method:**

This was a cross-sectional study. Four hundred sixteen students from medicine, physiotherapy, pharmaceutical sciences, medical laboratory sciences, nursing and pharmacy departments participated. Data were collected using an adapted Nordic Musculoskeletal Questionnaire. Participants were systematically and randomly selected from the respective class lists. Data were analysed using descriptive statistics and a Chi-squared test for association. Authorisation was obtained from the Dean of Students’ office and the Physiotherapy Department.

**Results:**

The response rate was 100%. Of the respondents, 57.5% (239) were male, and 42.5% (177) were female. The majority, 88.5% (368), were aged between 18 and 25. The overall prevalence of MSDs was 75.7% (315), with specific conditions reported as follows: low back pain at 59% (187), neck pain at 40.7% (129), shoulder pain at 33.1% (101) and upper back pain at 31.9% (101). Physiotherapy students (87.2%, 41) and pharmaceutical sciences students (87.2%, 34) were the most affected. Associated factors included physical injury (*p* = 0.000), year of study (*p* = 0.017), psychological distress (*p* = 0.006), long lectures (*p* = 0.013) and clerkship (*p* = 0.023).

**Conclusion:**

Health professional students are at high risk of developing MSDs, and preventive measures must be considered.

**Clinical implications:**

These findings will guide the development of a prevention protocol for MSDs and counselling for health professional students at the university.

# Abstract: An overview of data to determine indicators for walking ability of middle cerebral artery infarcts 3 months post-stroke

**Published:** 2024-11-29

**Authors:** Retha Nienaber

**Affiliations:** Rehabworx Groenkloof Hospital, Pretoria, South Africa

## Abstract

**Background:**

Walking ability data on ischaemic and haemorrhagic strokes in South Africa is limited. To be able to guide therapists in long-term planning for patients, data collected on ischaemic Middle Cerebral Artery (MCA) strokes in an acute private hospital in Pretoria, Gauteng, was reviewed to be able to assist with goal setting and future recommendations with regards to the patient’s walking ability.

**Objectives:**

To determine indicators for walking ability of middle cerebral artery infarcts 3 months post-stroke.

**Method:**

Stroke data were collected at an acute hospital in Pretoria from 01 July 2015 to 31 October 2023. Ischaemic stroke data were collected according to the arterial distribution of the strokes, as confirmed by the treating neurologist, focussing on middle cerebral artery infarcts. Outcome measures included the National Institutes of Health Stroke Scale (NIHSS), the premorbid Modified Rankin Scale (MRS) and the MRS at 3 months follow-up. A two-step cluster analysis was conducted to determine if meaningful groupings (clusters) of patients exist.

**Results:**

During our study period, 872 patients were admitted with an ischaemic infarct, and 479 were diagnosed with an MCA infarct – about 72% of the patients presented with their first-ever stroke. The mean age of all the subjects was 69 years. The male population group was 51%. The average length of stay for the MCA infarcts was 11 days. The cluster analysis identified two clusters, with walking ability as the most important indicator, with 286 patients in the ‘walking’ cluster and 160 in the ‘non-walking’ cluster.

**Conclusion:**

An admission with a lower NIHSS score, premorbid MRS of 0, age, being male and having a left MCA showed indicators for walking ability 3 months after an MCA infarct.

**Clinical implications:**

Patient functional status at admission, gender and side of stroke are important factors for patient mobility at 3 months post MCA stroke.

# Abstract: Overview of mobility data collected on Parkinson’s patients completing the Lee Silvermann Voice Treatment-BIG (LSVT-BIG®) programme

**Published:** 2024-11-29

**Authors:** Retha Nienaber

**Affiliations:** Rehabworx Groenkloof Hospital, Pretoria, South Africa

## Abstract

**Background:**

Patients living with Parkinson’s disease (PD) face several mobility limitations on impairment, activity and participation level, according to the International Classification of Function, Disability and Health. To establish if the LSVT BIG^®^ programme impacts patients living with PD mobility and balance, data were captured on different mobility outcome measures at a rehabilitation setting in Pretoria, Gauteng, from which various conclusions have been drawn.

**Objectives:**

To establish the influence of the LSVT BIG^®^ programme on patients with PD.

**Method:**

Data were collected between April 2019 and February 2024 at an outpatient rehabilitation setting in Pretoria. Patients were included in our study if diagnosed with PD (and optimally medically managed) by a neurologist and had completed 16 LSVT BIG^®^ therapy sessions delivered by a trained LSVT BIG^®^ clinician. Data were collected pre- and post-the four consecutive week programme (completing 16 sessions) on the 10m walk test (10MWT), the Berg Balance Scale (BBS), five Times Sit-stand (5xSTS), Timed Up and Go test (TUG), Timed Up and Go-cognitive (TUG-cognitive) and Timed up and Go-manual (TUG-manual). A paired differences non-parametric test determined statistical differences between the pre and post-scores.

**Results:**

Eleven subjects completed 16 LSVT BIG^®^ therapy sessions in four consecutive weeks of which eight were males. The mean age was 66. The results showed statistically significant differences (*p* < 0.05) between the pre and post-BBS, TUG, TUG-cog and TUG-manual scores.

**Conclusion:**

Patients living with PD significantly improved their balance and TUG scores, thus reducing their risk of falling and improving their quality of life.

# Abstract: Same-day discharge after early mobilisation and increased frequency of physiotherapy following hip and knee arthroplasty

**Published:** 2024-11-29

**Authors:** Retha-Mari Prinsloo, Monique M. Keller

**Affiliations:** Department of Physiotherapy, Faculty of Health Science, University of the Witwatersrand, Johannesburg, South Africa

## Abstract

**Background:**

Advanced rehabilitation pathway (ARP) after hip and knee arthroplasties is gaining ground in South Africa. A multidisciplinary team in Rustenburg, South Africa, implemented an ARP with the first same-day discharge (SDD) from the hospital. The lack of evidence of physiotherapy protocols within an ARP determined our study.

**Objectives:**

Our study determined and compared hospital length of stay (LOS) in hours, patient satisfaction using the Western Ontario and McMaster Universities Osteoarthritis Index (WOMAC), and patient safety as measured by 30-day readmission rates and costs between the cohorts.

**Method:**

A quantitative prospective patient (treatment *n* = 60) group receiving early mobilisation with increased frequency of physiotherapy on postoperative day zero (POD0) was compared to a conservatively managed retrospective historical (control *n* = 60) group following post-operative elective hip and knee arthroplasties.

**Results:**

The prospective group which were significantly improved relative to the retrospective group included decreased LOS (median 7.650, *p* < 0.001), less pain at 6 weeks (mean 16.20, standard deviation [s.d.] = 2.673, *p* < 0.001), less stiffness (mean 5.82, s.d. = 1.214, *p* = 0.007), higher function (mean 54.87, s.d. = 8.544, *p* < 0.001), lower hospital cost (mean R43340, *p* < 0.001), physiotherapy cost (mean R1069, *p* < 0.001) and total costs compared to the retrospective group (mean R117062, *p* < 0.001).

**Conclusion:**

Safe and cost-effective SDD is possible in an ARP with earlier mobilisation and increased frequency of physiotherapy on POD0.

**Clinical implications:**

Achieving safe SDD after hip and knee arthroplasty saved costs and improved patient satisfaction, with decreased LOS being beneficial for medical funders and stakeholders, including the government, aiming to implement National Health Insurance.

# Abstract: Returning to running postpartum: A scoping review

**Published:** 2024-11-29

**Authors:** Rochelle Bouwer, Corlia Brandt

**Affiliations:** Department of Physiotherapy, Faculty of Health Science, University of the Witwatersrand, Johannesburg, South Africa

## Abstract

**Background:**

Women returning to running postpartum lack comprehensive, context-specific guidelines.

**Objectives:**

Our study aimed to systematically explore existing evidence and guidance for returning to running after childbirth.

**Method:**

Joanna Briggs Institute (JBI) methodology for a scoping review was employed using a three-step search strategy on PubMed, CINAHL and Scopus, including only English sources. The initial search was conducted on 12 December 2022, and repeated on 13 December 2023. Two independent reviewers conducted two screening phases, supplemented by reference list reviews and stakeholder contacts. Thematic and content analysis was performed.

**Results:**

Our study included 30 sources with overall findings highlighting the need for more primary research in clinical settings. Less than half (11) were identified as primary research articles, while the majority (18) were grey literature. Only two sources collected data in a clinical setting. The majority (24) offered guidance and education for postpartum runners (PPRs) or medical staff assisting them. Other aspects included biomechanical changes of PPRs through clinical tests and running analysis, while questionnaires explored outcomes such as risk factors for pain, urinary incontinence and pelvic floor dysfunction. Running behaviours in PPRs were also assessed using questionnaires.

**Conclusion:**

All sources were published within the last decade, underscoring the novelty of this research area. The findings emphasise the need for further research to address existing gaps and enhance understanding in this field.

**Clinical implications:**

Research on returning to running postpartum is crucial for guiding women to a safe return to running in this period and to mitigate injuries and complications.

# Abstract: How do women in South Africa return to running postpartum

**Published:** 2024-11-29

**Authors:** Rochelle Bouwer, Corlia Brandt

**Affiliations:** Department of Physiotherapy, Faculty of Health Science, University of the Witwatersrand, Johannesburg, South Africa

## Abstract

**Background:**

A gap exists in evidence-based guidelines for women returning to running after childbirth, leading to inconsistencies in guidance given to postpartum runners (PPRs). Insight into the behaviour of PPRs will assist in furthering knowledge towards informed guidelines.

**Objectives:**

Our study aimed to explore the general practices among PPRs in South Africa.

**Method:**

A convenience sample of 241 female recreational and competitive PPRs in South Africa completed an online survey hosted on RedCap. Data included demographics, childbirth history, postpartum strength training and return to running practices. Adherence to guidelines, pelvic floor dysfunction and injuries were also recorded. Descriptive and inferential statistics were used to analyse the data (α = 0.05).

**Results:**

The median age was 35, with the majority being Caucasian (66.4%) and recreational runners (94.9%) who engaged in road running (91.9%). Postpartum runners resumed running at a median of 12 weeks postpartum without specific guidelines, while 60.26% did not engage in post-childbirth strengthening exercises. Postpartum runners self-determined their running distance and frequency, initially following a run/walk programme. Pelvic heaviness/dragging was reported by 25.6%, and only 19.5% reported injuries after returning to running.

**Conclusion:**

The general practices established in our study enhance understanding of the behaviour and abilities of PPRs, which can assist healthcare professionals and direct guideline development.

**Clinical implications:**

Healthcare professionals should tailor return to sport guidelines for PPRs. Further research must explore associations between postpartum behaviours and their impact on running performance.

# Abstract: Intra-pleural drainage in patients with a chest tube following trauma – The importance of timely physiotherapy

**Published:** 2024-11-29

**Authors:** Ronel Roos, Jocelyn Kirsty Finn

**Affiliations:** Department of Physiotherapy, Faculty of Health Sciences, University of the Witwatersrand, Johannesburg, South Africa

## Abstract

**Background:**

Tube thoracostomy is used to manage intra-pleural abnormality in patients and the presence of this device may affect patients’ mobility and ease of breathing.

**Objectives:**

The purpose of our study was to establish the physiotherapy treatment modalities and outline the effects on patients’ pain, respiratory function, mobility and exercise capacity. Secondary outcomes reviewed included length of hospital stay (LOS), duration of intra-pleural drainage and rate of infection.

**Method:**

An effective systematic review using the PICO approach and JBI SUMARI was carried out. A step-by-step search strategy of PubMed, CINAHIL Plus, the Cochrane Library, Physiotherapy Evidence Database (PEDro), Scopus, Science Direct and Google Scholar was completed. A meta-analysis was conducted for early versus late chest physiotherapy implementation when reviewing the impact on hospital LOS and duration of intra-pleural drainage.

**Results:**

Six studies were included for final review that resulted in *n* = 596 study participants of which most were men (*n* = 338, 56.7%) between the ages of 27 and 32 years old. Proprioceptive neuromuscular facilitation assisted with pain reduction and improved trunk range of motion and thoracic expansion. Continuous positive airway pressure ventilation, respiratory physiotherapy, upper limb and trunk exercises as well as ambulation reduced the duration of intra-pleural drainage, hospital LOS and pulmonary complications.

**Conclusion:**

Early combination therapy is an important management strategy for treating patients with intra-pleural abnormalities.

**Clinical implications:**

Few studies currently identify and review physiotherapy treatment modalities and as such, a need exists for further clinical trials to be conducted in this area of physiotherapy.

# Abstract: Upper limb muscle strength and exercise endurance as predictors of successful extubation in mechanically ventilated patients

**Published:** 2024-11-29

**Authors:** Caroline R. Brandon, Danielle Fourie, Stefan Kemp, Omphile Makhafola, Londiwe Magubane, Saheera Stevens, Caelin van der Westhuizen, Martins Nweke, Rozelle Labuschagne

**Affiliations:** Department of Physiotherapy, Faculty of Health Sciences, University of Pretoria, Pretoria, South Africa

## Abstract

**Background:**

Avoiding failed extubation is paramount; therefore, predictors for successful extubation are paramount.

**Objectives:**

Our study aimed to determine if physiotherapists can use upper limb muscle strength and exercise endurance as predictors for successful extubation in mechanically ventilated patients. The statistical objective was to develop a prediction equation based on upper limb muscle strength and exercise endurance for the outcome of extubation.

**Method:**

A total of 57 patients were eligible for evaluation in an academic hospital’s surgical and medical intensive care units (ICUs) in South Africa. Peripheral and respiratory muscle strength were evaluated using the Oxford grading scale, Medical Research Council score (MRC-score), handgrip dynamometer and maximum inspiratory pressure (MIP). Exercise endurance was tested while patients rode the MOTOmed^®^ letto2 cycle ergometer for 6 min with the upper limbs.

**Results:**

Exercise endurance (time the patient rode actively) (*p* = 0.005), general body muscle strength (MRC-score: *p* = 0.007) and number of days ventilated (*p* = 0.005) were associated with successful extubation. The newly developed prediction equation comprised of exercise endurance and the number of days ventilated exhibits a sensitivity of 81.82% and a specificity of 77.14% to predict successful extubation.

**Conclusion:**

Exercise endurance incorporated in an algorithm shows excellent potential to predict successful extubation.

**Clinical implications:**

Physiotherapists can predict successful extubation using the newly developed prediction equation of exercise endurance and number of ventilated days. Successful extubation may reduce the length of stay in the ICU and financial expenditure and increase the patient’s functional ability post-ICU discharge.

# Abstract: Breathing pattern dysfunction in patients with median sternotomy following hospital discharge: A cross-sectional study

**Published:** 2024-11-29

**Authors:** Samantha Lee Anselmo Hurst, Ronel Roos

**Affiliations:** Department of Physiotherapy, Health Sciences, University of the Witwatersrand, Johannesburg, South Africa

## Abstract

**Background:**

Patients following cardiac surgery via median sternotomy experience a deficit in chest expansion and respiratory muscle strength during hospital stay. To date, no study has assessed whether there is long-term breathing pattern dysfunction (BPD) in patients.

**Objectives:**

Our study aimed to establish whether patients who are 3 months to 1 year post-surgery still experience BPD and to identify the risk factors for the development of this dysfunction.

**Method:**

A cross-sectional observational study was conducted. Fifty-two participants between the ages of 18 and 65 years participated after hospital discharge via a telephonic video consultation. Outcome measures included the Physical Activity Vital Signs, demographic and BPD-related questionnaires, chest expansion and the Breath Hold Time (BHT) test. Data were analysed using descriptive and inferential statistics with significance set at *p* ≤ 0.05.

**Results:**

Most participants (51.90%, *n* = 27) scored positive in three outcome measures relating to BPD. Risk factors for the development of BPD included female gender, ‘other’ type of surgery, cardiac complications and return to work rate. The amount of weekly aerobic exercise after discharge and the absence of postoperative complications during hospital stay reduced the risk of BPD.

**Conclusion:**

There is a high prevalence of long-term BPD in patients after cardiac surgery via median sternotomy.

**Clinical implications:**

Assessment of BPD among patients post-cardiac surgery should consider the multidimensional nature of the dysfunction. Furthermore, early identification and management of risk factors should take place. Additionally, aerobic exercise positively influenced all dimensions of BPD and should, therefore, be encouraged.

# Abstract: Lifestyle interventions in comorbid mental and physical illness: A systematic review

**Published:** 2024-11-29

**Authors:** Sandy Lord, Vaneshveri Naidoo, Karien Mostert

**Affiliations:** Department of Physiotherapy, Health Sciences, University of the Witwatersrand, Johannesburg, South Africa

## Abstract

**Background:**

Patients with mental health disorders (MHDs) often present with chronic illness and complain of pain and poor physical health. They present with a high burden of disease and poor quality of life. Lifestyle interventions are cost-effective strategies which seem to be an effective adjunct in managing MHDs and chronic illness.

**Objectives:**

Our study aimed to determine the effectiveness of lifestyle interventions on health-related quality of life in patients presenting with both mental and physical health disorders.

**Method:**

The systematic review used the Joanna Briggs Institute (JBI) methodology. MEDLINE (Ovid), CINAHL (EBSCO), LiLACS, Scopus, PEDro and Cochrane Central Register of Controlled Trials were searched to identify published randomised control studies available in English from 2011 to 2022. The inclusion criteria were adults with comorbid physical and MHDs who received lifestyle interventions (including stress management strategies) to improve health-related quality of life. Group interventions were excluded from our review.

**Results:**

A total of four studies were included in our review. The primary lifestyle intervention discussed was cognitive behavioural therapy (CBT), with one study incorporating physical activity. While CBT was effective in managing patients with depression, anxiety and comorbidities such as obesity, diabetes and heart failure, the benefits were not sustained in the long term.

**Clinical implications:**

Stress management modalities such as CBT may be effective in the shorter term to help manage patients with comorbid physical and MHDs. However, these studies were limited to individual therapies. Studies with group activities were excluded, which excluded a large range of other lifestyle interventions.

# Abstract: The impact of a brief online education intervention on qualified physiotherapists’ knowledge, attitudes and beliefs about chronic low back pain

**Published:** 2024-11-29

**Authors:** Sandy Lord, Lara Davis, Benita Olivier, Lorraine Jacobs-Scott

**Affiliations:** 1 Department of Physiotherapy, Health Sciences, University of the Witwatersrand, Johannesburg, South Africa; 2 Lorraine Jacobs Physiotherapy, Johannesburg, South Africa

## Abstract

**Background:**

Low back pain (LBP) remains a major global disability concern. Recent literature recommends using stratification tools based on psychosocial factors, followed by tailored treatment for risk-category patients.

**Objectives:**

This approach aims to prevent chronicity and reduce the LBP burden.

**Method:**

In a quantitative pre-post survey analysis, data collected between 2016 and 2020 from 536 participants in a three-part webinar series were analysed. Physiotherapists’ knowledge, attitudes and beliefs pre- and post-intervention were measured using the Pain Attitudes and Beliefs Scale for Physiotherapists (PABS-PT) and the Neurophysiology of Pain Questionnaire (NPQ). The data collected before and after the webinar series were compared using descriptive analysis, dependent t-tests and Pearson correlation analyses.

**Results:**

Baseline PABS-PT scores were 53.74 ± 12.80 (44.8%) for the biomedical subscale and 35.52 ± 4.65 (65.8%) for the biopsychosocial subscale. The baseline NPQ mean score was 78.4%. The biomedical treatment orientation decreased by 8.8% following the educational intervention, while biopsychosocial attitudes and beliefs strengthened by 7.6%. Pain neuroscience knowledge improved by 6.6%. Notably, enhanced biopsychosocial orientation correlated with reduced biomedical focus post-intervention.

**Conclusion:**

The findings of our study reflect that a targeted educational intervention may assist in modifying the attitudes and beliefs of physiotherapists regarding their role in adequately assessing and managing people with longstanding pain and disability towards a biopsychosocial framework.

**Clinical implications:**

The significance of this result is that pain education may be useful in augmenting the knowledge and understanding of physiotherapists regarding low back pain, which could promote better adherence to clinical guidelines and improve patient outcomes.

# Abstract: Public-private partnerships to enhance access to quality rehabilitation services in KwaZulu-Natal ahead of National Health Insurance implementation: Stakeholders’ perspectives

**Published:** 2024-11-29

**Authors:** Senzelwe Mazibuko, Thatananthee Nadasan, Pragashnie Govender

**Affiliations:** Department of Physiotherapy, Faculty of Health Sciences, University of KwaZulu-Natal, Durban, South Africa

## Abstract

**Background:**

Rural South African provinces, including KwaZulu-Natal (KZN), struggle with limited access to quality rehabilitation services. While public-private partnerships (PPPs) are favoured for public health delivery, their role in rehabilitation remains unclear.

**Objectives:**

We explored PPPs’ potential to enhance access, especially amid National Health Insurance (NHI) implementation.

**Method:**

In this mixed-methods study conducted across three districts in KZN, 42 rehabilitation practitioners, including physiotherapists, completed a survey questionnaire. Alongside the survey, an additional 15 managers (rehabilitation managers, provincial rehabilitation managers and representatives from the provincial office of social development) were included in focus group discussions and interviews. Descriptive and thematic analyses were conducted, and findings were reported.

**Results:**

National Health Insurance knowledge was marginal (Median = 3.0, IQR = 1.0), but general perceptions and sentiment around NHI were less optimistic, with some scepticism around its feasibility and the lack of specific emphasis on rehabilitation as a mechanism for improving the health system. As a result, the overall NHI perceptions score was lower (Median = 2.3). Participants’ understanding of PPPs was predominantly positive (Median = 3.0, IQR = 0.3), as was the general sentiment on PPPs, as evidenced by the practitioners tending to disagree with the notion that ‘PPPs cannot improve the availability of rehabilitation services because public health is too dysfunctional’ (Median = 2.0, IQR = 1.0).

**Conclusion:**

Participants exhibit limited understanding of PPP but are well-versed in NHI.

**Clinical implications:**

The findings underscore the need for enhanced education and policy emphasis on the role of rehabilitation within the NHI scheme. Additionally, promoting awareness and understanding of PPPs can potentially improve rehabilitation service delivery and access within the healthcare system.

# Abstract: Physical activity interventions to reduce non-communicable diseases in older people in sub-Saharan Africa

**Published:** 2024-11-29

**Authors:** Shane Naidoo, Nirmala Naidoo

**Affiliations:** Department of Health and Rehabilitation Sciences, Faculty of Health Sciences, University of Cape Town, Cape Town, South Africa

## Abstract

**Background:**

Sub-Saharan Africa (SSA) has followed global trends with an escalation in the population growth of older people (OP). Older people have a high prevalence of non-communicable diseases (NCDs) that are indirectly correlated with physical activity (PA). Physiotherapists are proficient partners in promoting healthy ageing by implementing PA interventions at individual, community and managemental levels.

**Objectives:**

Explore the extent and effectiveness of PA interventions implemented in SSA to assist with managing NCDs in OP.

**Method:**

Our review synthesised randomised controlled trials (RCTs) published in English after 2010 on PA interventions implemented to manage NCDs on OP in SSA. The JBI methodology for scoping reviews was effectuated with PubMed, EBSCOhost, Scopus and ProQuest searches.

**Results:**

Sixty-seven RCTs met the inclusion criteria for synthesis that trialled interventions of supervised structured PA (*n* = 30), education on PA and lifestyle behaviour (*n* = 30) and structured PA interventions combining education on lifestyle behaviour (*n* = 7). There were studies with clinical statistically significant (*p* < 0.05) outcomes post-intervention for blood pressure (*n* = 48), cardiac parameters (*n* = 37), blood sugar levels (*n* = 15), body composition (*n* = 10), motor function (*n* = 13), health-related quality of life (*n* = 8) and cognition (*n* = 2).

**Conclusion:**

Our review presents a comprehensive overview of how structured PA programmes, either with or without an educational component on lifestyle changes, effectively address NCDs in OP from SSA.

**Clinical implications:**

To direct clinicians in implementing contextual evidence-based PA interventions when addressing NCDs and their risk factors in OP, hence facilitating favourable outcomes.

# Abstract: Effectiveness of a structured aerobic exercise intervention for adults living with HIV and AIDS on highly active antiretroviral therapy: A single-blinded randomised control trial

**Published:** 2024-11-29

**Authors:** Tania Steyl, Merly Sajan, Adedapo Wasiu Awotidebe, Joliana Phillips

**Affiliations:** Department of Physiotherapy, Community and Health Sciences, University of the Western Cape, Cape Town, South Africa

## Abstract

**Background:**

A high prevalence of human immunodeficiency virus (HIV)-associated disability, including impairment in mobility, is associated with reduced quality of life (QoL). Improving QoL is particularly salient for physiotherapists, given their holistic approach to providing care for people living with HIV or AIDS (PLWHA). The authors report that exercise is a key management strategy for improving physical function and QoL in different patient cohorts.

**Objectives:**

The aim is to determine the effectiveness of aerobic exercise on physical function and QoL in PLWHA on Highly Active Antiretroviral Therapy (HAART).

**Method:**

Simple randomisation was used in a single-blinded randomised controlled trial involving 60 participants who met the inclusion criteria and consented to participate. Thirty participants in the intervention group completed an 8-week aerobic exercise intervention, while 30 participants in the control group received standard care. The following outcome measures were assessed: anthropometric measurements, cardiopulmonary indices, the Simmonds battery of functional tests and the WHOQoL-BREF. Inferential statistics were employed to compare baseline variables both between and within groups as well as post-treatment outcomes. The significance level was set at 0.05.

**Results:**

Within the intervention group, median scores for all components of QoL, maxHR, VO_2_max, 6MWT and balance increased from pre-intervention to post-intervention. The intervention group had a significantly higher median score (8.50) than the control group (7.00) on overall quality of life (Mann–Whiney U = 175.00, z = 4.18, *p* < 0.001) with a large effect size (r = 0.76).

**Conclusion:**

Aerobic exercise could potentially improve physical function and QoL of PLWHA.

**Clinical implications:**

Aerobic exercise is a viable and inexpensive therapeutic treatment option that should be included in the daily management of PLWHA.

# Abstract: International students need physical activity aligned with global targets against noncommunicable and mental diseases

**Published:** 2024-11-29

**Authors:** Nivyimana Willy, Zhou Daan, Mbasani Rogious, Djivoh S. Yollande, Bayumvire Claudette

**Affiliations:** 1 Department of Rehabilitation medicine and physical therapy, Jinzhou medical university, Jinzhou, China; 2 Department of Rehabilitation, The Third Affiliated Hospital of Jinzhou Medical University, Jinzhou, China; 3 Department of Clinical Sciences, Faculty of Physiotherapy and Rehabilitation School, National Institute of Public Health, Bujumbura, Burundi; 4 Rakai General Hospital, Rakai, Uganda; 5 Department of Physical Medicine and Rehabilitation, Hubert Koutoukou MAGA National University Hospital Center, Abomey-Calavi University, Cotonou, Benin

## Abstract

**Background:**

The prevalence of noncommunicable diseases and mental problems is increasing worldwide. Physical inactivity is the second risk factor for noncommunicable diseases, while physical activity enhances mental well-being. International students are at risk of noncommunicable diseases and mental disorders because of multiple challenges.

**Objectives:**

Our study aimed to assess the relationship between physical activity with mental health and cardiometabolic risk factors among international students in China.

**Method:**

A cross-sectional study was conducted using an online questionnaire comprising cardiometabolic characteristics, International Physical Activity Questionnaire Short Form, Generalised Anxiety Disorder, Patient Health Questionnaire and the Pittsburgh Sleep Quality Index. Data were analysed using descriptive statistics, independent t-tests and linear regression.

**Results:**

Our results found that 8.1%, 3.8% and 2.5% of subjects self-reported hypertension, hyperlipidaemia and hyperglycaemia, respectively. The prevalence of low physical activity, depression, anxiety and poor sleep quality were 71%, 61.2%, 53.7% and 63.7%, respectively. Participants without hypertension, hyperlipidaemia and hyperglycaemia performed significant Metabolic Equivalents (METs)/week in the overall type of exercises (*p* < 0.01) than their peers. Moderate to high levels of physical activity were negatively associated (*p* < 0.01) with anxiety, depression and sleep disorders.

**Conclusion:**

Our study indicated a negative association between physical activity levels with anxiety, depression and sleep disorders. Participants without cardiometabolic risk factors were more active.

**Clinical implications:**

To meet one of the nine global targets of improving the prevention and treatment of non-communicable diseases and mental health disorders by reducing the prevalence of insufficient physical activity by 15% by 2030, our study suggests enhancing physical activity in vulnerable populations like international students whose numbers are increasing every year.

# Abstract: Namibia supports a century of rehabilitation and beyond. A first in Namibia: Reflection on the development of the first Namibian Physiotherapy Education Programme

**Published:** 2024-11-29

**Authors:** Zenra L. Buys, Tonderai W. Shumba, Etheline O. Geurtze

**Affiliations:** Department of Occupational Therapy and Physiotherapy, Faculty of Health Sciences and Veterinary Medicine, University of Namibia, Windhoek, Namibia

## Abstract

**Background:**

To contribute to a developing healthcare system through education, the first physiotherapy educational programme was established in 2018 to address the rising need for rehabilitation services in warm and beautiful Namibia.

**Objectives:**

Our study reflects on the journey since the intake of students and celebrates a century of rehabilitation with our neighbouring African countries.

**Method:**

The curriculum was developed through technical working groups of the University of Namibia (UNAM), the Namibian Society of Physiotherapy (NSP) and the Motor Vehicle Accident Fund (MVA). The first 2 years of inception had two full-time, two part-time and occasional visiting lecturers from Stellenbosch University. In 2022, further stakeholder consultation was held to transform the curriculum.

**Results:**

The programme was accredited in 2021 for 3 years. In 2023, a new, transformed curriculum was implemented for the first-year cohort. The programme has celebrated three cohorts of graduates (28 physiotherapists), now employed in Namibia. The staff complement has risen to eight full-time staff.

**Conclusion:**

Namibia has made remarkable leaps in ensuring rehabilitation is central to health service delivery, aligning itself with the vision of the World Health Organization. We extend our appreciation to the South African Physiotherapy academia that has assisted with programme development, moderation of examinations and external examination. The impact of their input will be assessed, as we are scheduled for a re-inspection of the programme in 2024.
